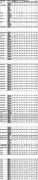# Biomarker confirmed reference values for acoustic voice features: findings from the Framingham Heart Study

**DOI:** 10.1002/alz70856_104922

**Published:** 2026-01-07

**Authors:** Huitong Ding, Xavier Serrano, Cody Karjadi, Christina B. Young, Elizabeth C. Mormino, Preeti Sunderaraman, Ashita S. Gurnani, Rhoda Au, Katherine A. Gifford

**Affiliations:** ^1^ Boston University Chobanian & Avedisian School of Medicine, Boston, MA, USA; ^2^ Stanford University School of Medicine, Stanford, CA, USA

## Abstract

**Background:**

Interest in acoustic voice features as digital biomarkers of underlying Alzheimer's disease (AD) has been increasing. However, lacking confirmation of AD specificity and reference values or normative information, particularly in relation to AD‐specific biomarkers, greatly limits the ability to determine measurement thresholds that are clinically meaningful. We present preliminary normative values of acoustic voice features for those who are positron emission tomography (PET) beta amyloid positive (Aß+) and negative (Aß‐).

**Method:**

This study included 268 cognitively unimpaired participants (mean age 57.2 ± 9.9 years; 50.4% female) from the Framingham Heart Study Brain Aging Program who had voice recordings of neuropsychological assessment obtained within one year before amyloid PET imaging. Sixty‐five acoustic features (i.e., prosodic, spectral, and sound quality voice features) were extracted from recordings during the Wechsler Memory Scale Logical Memory Delayed recall tests using open‐source Speech and Music Interpretation by Large‐space Extraction (OpenSMILE). Reference values were established at the 2.5th, 25th, 50th, 75th, and 97.5th percentiles for each acoustic feature within the entire sample, amyloid‐positive (Aß+) and amyloid‐negative (Aß‐) groups. Differences between the Aß+ and Aß‐ groups were evaluated using Mann‐Whitney U tests.

**Result:**

Of the 268 participants, 30 (11%) were Aß+. Reference values for all 65 acoustic features were established across all percentile thresholds within the whole sample, the Aß+ and Aß‐ groups (see Table). Four acoustic features differed between the Aß+ and Aß‐ groups: voicingFinalUnclipped (*P* = 0.03), pcm_fftMag_spectralKurtosis (*P* = 0.04), MFCC[5] (*P* = 0.02), and MFCC[10] (*P* = 0.03). Three of them have higher median values in Aß+ group. As a sound quality measure, VoicingFinalUnclipped indicates the voicing probability of the final fundamental frequency candidate without zero‐clipping. The pcm_fftMag_spectralKurtosis represents magnitude of spectral kurtosis. MFCCs reflect the power spectrum of a sound and are mathematical representations of essential human speech characteristics.

**Conclusion:**

These results suggest acoustic features may be an effective marker for preclinical AD screening of older adults who are Aß+. Future studies should stratify based on biomarker status to refine reference values and expand doing so with more diverse populations.